# Effect of Silkworm Pupa Protein Hydrolysates on Proliferation of Gastric Cancer Cells In Vitro

**DOI:** 10.3390/foods11152367

**Published:** 2022-08-07

**Authors:** Weixin Li, Lixia Mu, Yuxiao Zou, Weifei Wang, Haifeng Zhao, Xuli Wu, Sentai Liao

**Affiliations:** 1 Sericultural & Agri-Food Research Institute Guangdong Academy of Agricultural Sciences/Key Laboratory of Functional Foods, Ministry of Agriculture/Guangdong Key Laboratory of Agricultural Products Processing, Guangzhou 510610, China; 2 School of Food Science and Engineering, South China University of Technology, Guangzhou 510640, China; 3 School of Public Health, Health Science Center, Shenzhen University, Shenzhen 518060, China

**Keywords:** silkworm pupa protein hydrolysates, proliferation inhibition, morphological observation, apoptosis and cycle

## Abstract

The proliferation inhibition effects of the hydrolysates from silkworm pupa proteins on MGC-803 gastric cancer cells were investigated in this study. The specific morphological changes (cell membrane, cell nucleus and cytoskeleton) of cells were measured. In vitro, the proliferation of MGC-803 cells was inhibited by silkworm pupa protein hydrolysates (SPPHs) in a dose-dependent manner. The flow cytometry analysis showed that the blocking effect of SPPHs on the MGC-803 cells was mainly in the G_0_/G_1_-phase. The morphological changes, disintegration of the cytoskeleton and retardant cell cycles were probably related to the activation of apoptosis. Thus, SPPHs could be promising as a chemopreventive agent due to their ability to promote apoptosis of tumor cells.

## 1. Introduction

Food-derived hydrolysates are some of the most widely used natural products in the world. They have been used extensively in the healthcare industry and also in modern medicine. Large numbers of cancer-preventive hydrolysates have been reported in recent years [1,2].

The silkworm pupa is well known as an efficient large-scale producer of silk thread. It is a sub-product obtained after the extraction procedure of silk threads and traditionally used as fertilizer, animal feed, food material and traditional medicine in some countries, such as China, Japan, Korea, India and Thailand [3]. The interest in the silkworm pupa has grown in the last decades on the basis of its putative nutritional function and pharmacological effects, which include antioxidant and anti-inflammatory activities [4,5]. Recently, SPPHs have been proved to have good performance against tumors that can help in the therapy and prevention of cancer patients with physical damage and in suppressing some possible inflammation factors [6].

Not only the actions of inhibiting cell proliferation and promoting apoptosis, but also other mechanisms have been proposed to explain the anti-tumor effect of SPPHs, such as anti-inflammatory and antioxidant activities [7,8]. Some researchers have tried to find the specific morphological changes of the apoptosis process. There is evidence showing that a large number of anticancer hydrolysates exhibit no cytotoxicity and trigger apoptosis by acting on cellular proteins, and the induced apoptotic process involves both intracellular and extracellular pathways [9]. However, the mechanism of inhibiting cell proliferation by SPPHs remains unclear.

Our earlier studies found that in the proliferation of HepG-2, A549 and MGC-803 cell lines, SPPHs could be specifically inhibited to the MGC-803 gastric cancer cell line in vitro [1,10,11]. However, the effect of the degree of hydrolysis (DH) and ultrafiltration on proliferation inhibition and apoptosis on MGC-803 cells had no relevant data. On the other hand, the results of specific morphological changes of MGC-803 cells were not provided. As such, our work will clarify the specific effects of SPPHs on cell morphological parameters and the possible effect on the cell cycles.

## 2. Materials and Methods

### 2.1. Chemicals and Reagents

Fresh silkworm pupa of Liangguang 2 (LG 2), a widely fed silkworm cultivar in Southern China, were purchased from the Guangzhou Huangsha seafood market (Guangdong, China). Buffer solutions PF-1, PF-2, PF-3, PF-4 and PF-RG of the amino acid determination system, a mixed standard solution of 38 kinds of physiological body fluid amino acids, ninhydrin and its buffer solution and other test reagents were purchased from Hitachi Co., Ltd. and Wako Pure Chemical Industries Co., Ltd. (Tokyo, Japan). MGC-803 gastric cancer cells were supplied by the National Infrastructure of Cell Line Resource (Beijing, China). DMEM (high glucose) culture medium, fetal bovine serum, 0.25% trypsin-EDTA (1X), penicillin–streptomycin and phosphate-buffered saline (PBS) were purchased from Gibco (New York, NY, USA). A Cell Counting Kit-8 (CCK-8) was purchased from Dojindo (Tokyo, Japan). Novozymes 37071 was purchased from Novozymes (Bagsvaerd, Denmark). Doxorubicin was purchased from QiYun Biological Technology Co., Ltd. (Guangzhou, China). A cell cycle and cell apoptosis detection kit (Product Code: C1052) and Annexin V-FITC Apoptosis Detection Kit (Product Code: C1062) were purchased from Beyotime (Shanghai, China). Beta tubulin antibody and fluorescein (FITC)–conjugated affinipure goat anti-mouse IgG (H + L) were purchased from Proteintech (Chicago, IL, USA). Phalloidin-TRITC and amino acid standard were purchased from Sigma (St. Louis, MO, USA). All other reagents used in this study were of analytical grade and purchased from Damao Chemical Reagent Factory (Tianjin, China).

### 2.2. Preparation of SPPHs and Determination of Free Amino Acids

#### 2.2.1. Preparation of SPPHs

The SPPH solution was prepared according to our previously reported method with minor modifications [1]. In brief, prior to enzymatic hydrolysis, the fresh silkworm pupa was dried at 60 °C for 48 h, then degreased by CO_2_ supercritical extraction treatment after drying and grinding through 60 mesh sieves. The optimum temperature of Novozymes 37071 protease is 50 °C; pH is adjusted to 9.0 with a PB-10 pH meter (Sartorius Corporation, Gottingen, Germany), then the amount of Novozymes 37071 protease is set at 5% of protein content. The hydrolysates with different DH can be obtained by controlling enzymolysis time with a pH-stat method [12]. After the reaction, solutions were heated at 100 °C for 5 min to inactivate the enzymes and centrifuged (CR 22GIII, HITACHI, Tokyo, Japan) at 4000× *g* for 30 min. The corresponding DH of the SPPH7~9 hydrolysates were 5%, 15% and 25%, respectively. Then, SPPH1~6 fractions were obtained from the SPPH7~9 hydrolysates via a pellicon compact ultrafiltration system and a TOO-300 peristaltic pump (Millipore, Burlington, MA, USA) with the molecular weight cutoffs (MWCOs) at 3k Da. According to the DH of the SPPH, the SPPH7 hydrolysates with molecular weight cutoffs (MWCOs) below 3k Da corresponded to SPPH1, while those with a molecular weight (Mw) over 3k Da belonged to SPPH4. SPPH2/SPPH5 and SPPH3/SPPH6 were obtained from SPPH8 and SPPH9 via the analogous method, respectively. All fractions were lyophilized in a freeze drier (Labconco Freezone 7948030, Labconco Corporation, Kansas, MO, USA) for 3 days and sealed at −20 °C.

#### 2.2.2. Determination of Free Amino Acids

The pre-treatment of samples and determination method of amino acids refers to the Hitachi L-8900 instructions [13] and Chen, L et al. [14]. A combination of 10 mL of 6% 5-sulfosalicylic acid solution and 0.50 g of activated carbon were added to the sample, then it was placed in the refrigerator overnight and then centrifuged at 10,000 r/min for 30 min, and diluted to 25 mL with 0.06 mol/L HCl solution. All the samples were filtered using a 0.45 μm membrane filter (Millipore, Billerica, MA, USA) before being tested. The packed column for free amino acid analysis was 4.6 mm × 60 mm (filler 2622), with pump 1 flow rate at 0.35 mL/min and pump 2 flow rate at 0.30 mL/min, column temperature at 60 °C, detector temperature at 130 °C, detection wavelength at 570 nm for the first channel and 440 nm for the second channel and an injection volume of 20 μL. The analysis time of each sample was about 115 min. the actual concentration of the samples needed to be converted according to the molar mass.

### 2.3. Cell Culture

MGC-803 cells were routinely cultured in DMEM containing 10% (*v*/*v*) fetal bovine serum, 100 U/mL penicillin and 0.1 mg/mL streptomycin in a humidified atmosphere containing 5% CO_2_ in a 37 °C incubator (3110 Series, Thermo, Waltham, MA, USA). The logarithmic growth phase cells were used for all the experiments, and the SPPHs were dissolved in serum-free medium for use.

### 2.4. Proliferation Inhibition of MGC-803 Cells

MGC-803 cells were inoculated on 96-well plates (1 × 10^5^ cells per well) and cultured for 12 h. Then, the medium was replaced by fresh medium with SPPHs at various concentrations (0.31, 0.63, 1.25, 2.50 and 5.00 mg/mL). A blank control group was set by treatment with 200 μL of fresh culture medium. After respective treatments, all the cells continued to culture for 24 h. Then, according to the kit instruction, 10 μL of CCK-8 solution was added to each well and incubated for another 2 h, and the absorbance values (A) were measured at the 450 nm wavelength by using a microplate reader (TECAN Infinite 200, TECAN, Männedorf, Switzerland) as soon as possible. The inhibiting rate (IR) can be calculated by the following formula.

IR (%) = [(A_c_ − A_s_)/(A_c_ − A_b_)] × 100%

A_s_: Treated samples groups (culture medium, cells, CCK-8, SPPHs).
A_c_: Blank control groups (culture medium, cells, CCK-8).
A_b_: Background groups (culture medium, CCK-8).

### 2.5. Morphologic Observation of MGC-803 Cells

#### 2.5.1. General Microscope

MGC-803 cells were inoculated on 6-well plates and cultured for 12 h. The treated samples were added with 1 mL of SPPH3 at the concentration of 0.31 mg/mL. A blank control group and Doxorubicin (0.001 mg/mL) were also provided. After finishing different treatments, the cells were incubated for an additional time (6, 12, 18 or 24 h), and each group was placed to observe the morphological changes under a Leica inverted microscope; the observed additions were obtained following the method described by Wolf, D.E et al. [15].

#### 2.5.2. Fluorescence Microscope

In order to investigate the in-situ apoptosis, a fluorescent staining method was used as per the guidance [16,17]. Culturing of MGC-803 cells and sample adding treatments were consistent with those mentioned in Section 2.5.1, and they continued to culture for 24 h. First, culture medium was removed from the 6-well plates and they were washed with PBS carefully. Secondly, the Annexin V-FITC binding buffer was added to each well of the 6-well plates at a volume of 195 μL. Then this was gently mixed after adding 5 μL of Annexin V-FITC and 10 μL of propidium iodide (PI) to the 6-well plates. The cells were incubated in a dark place for another 10–20 min at the room temperature, then placed in an ice bath during the subsequent time. All the observation was completed within an hour under a fluorescent microscope (Leica, Weztlar, Germany).

#### 2.5.3. Laser Scanning Confocal Microscopy (LSCM)

Observation methods were conducted according to a previously reported protocol [18,19] and divided into the following two steps.

Making cell slides: The pretreatment of cells and sample adding treatments were the same as in Section 2.5.1, but a cell crawling piece was placed in each well of the 6-well plates before the inoculation of cells. The cells were cultured for an additional 24 h after completing the steps involved.

Cell fixation and labeling: Cell slides were cleaned with PBS three times, fixed with 4% paraformaldehyde for 20 min and washed three times again with 0.2% Triton X-100. Then, 5% horse serum was used to seal the membrane for 1 h and it was rinsed with 0.2% Triton X-100 three times. Beta Tubulin Antibody and Goat Anti-Mouse IgG (H + L) fluorescent antibody were added to label cell microtubules according to the reagent instructions. After that, they were washed three times with 0.2% Triton X-100. Then, Phalloidin-TRITC was directly added to those cell slides which were labeled with microtubules at a volume of 100 μL and they were incubated for 30 min at room temperature and washed three times again with 0.2% Triton X-100. Then, 100 μL of DAPI was added to the cell slides so as to stain the nucleus. After rinsing three times, gold antifade reagent was added to the cell slides to make a permanent slide, then the cytoskeleton was observed by LSCM (LSCM 710, Zeiss, Oberkochen, Germany).

### 2.6. Apoptosis

The pretreatments of the cells were consistent with Section 2.5.1, and a series of concentrations (0.02, 0.04, 0.08, 0.16 and 0.31 mg/mL) of SPPH3 were applied. The concentration of the SPPH1 and SPPH2 was 0.31 mg/mL. A blank control group and Doxorubicin (0.001 mg/mL) were also provided. After the completion of apoptotic stimulation, 5.0 × 10^4^~1.0 × 10^5^ cells were collected by centrifuge (5702R, Eppendorf, Hamburg, Germany) at 1000× *g* for 5 min. A total of 195 μL Annexin V-FITC Binding Buffer, 5 μL Annexin V-FITC and 10 μL PI were added, then the cells were gently resuspended. The cells were incubated in the dark for 10–20 min at room temperature. They was placed in the ice bath and detected by flow cytometry (BD Biosciences, San Jose, CA, USA) within an hour.

### 2.7. Cell Cycle Assay

Cell culture and dose treatments are identical to those mentioned in Section 2.6, and the cells were digested with trypsin, then centrifuged at 1000× *g* for 5 min to obtain the precipitated cells. The cells were resuspended with approximately 1 mL of PBS, then transferred to a 1.5 mL centrifuge tube and fixed for 12 h with 70% ethanol. The cells were centrifuged again after the fixation. According to the instructions, 0.5 mL PI staining solution was added to each sample. After staining, the fluorescence was detected at 488 nm wavelength by flow cytometry to evaluate the DNA content.

### 2.8. Statistical Analysis

Results were represented by means ± standard deviation (SD). Statistical analyses were performed by using the IBM SPSS Statistic Version 20.0 (SPSS Inc., Chicago, IL, USA). Data were subjected to ANOVA followed by Duncan to identify differences between values. A *p*-value less than 0.05 was considered statistically significant.

## 3. Results

### 3.1. Content of Free Amino Acids

**Figure 1:**
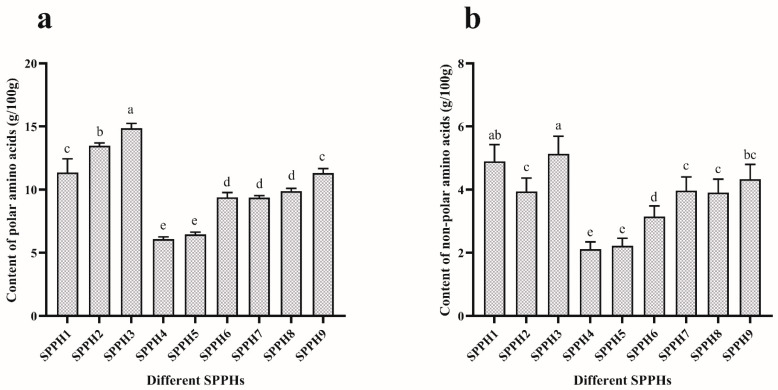
Amino acid content of SPPHs. (**a**) The content of polar amino acids in SPPHs. (**b**) The content of non-polar amino acids in SPPHs. All the data were analyzed by Duncan’s multiple comparisons; bars with no letter in common are significantly different at the level of *p* < 0.05.

In the previous study [11], we carried out a preliminary determination of the Mw distribution range of the SPPHs by HPLC, in which the component content of Mw > 5k Da was 18.32% and the component content of Mw < 5k Da was 81.68%. At present, we continue to classify and analyze amino acids in combination with the determination results of free amino acids. In general, the content of polar or non-polar amino acids in the SPPHs was positively correlated with the increase in DH. There is no doubt that deep hydrolysis contributes to the release of amino acids. For polar amino acids, as shown in Figure 1a, the SPPH4 (Mw > 3k Da, DH = 5%) component was the lowest, with a content of 6.08 ± 0.15 g/100 g, and the SPPH3 (Mw < 3k Da, DH = 25%) component was the highest, with a content of 14.87 ± 0.30 g/100 g. For non-polar amino acids, as shown in Figure 1b, the SPPH4 component was also the lowest, with a content of 2.11 ± 0.19 g/100 g, and the SPPH3 component was the highest, with a content of 5.31 ± 0.78 g/100 g. Analysis of the data revealed that the total amount of polar amino acids in the enzymatic solution was greater than the total amount of non-polar amino acids. Moreover, both the total amounts of polar and non-polar amino acids were positively correlated with the DH of the SPPHs. On the other hand, the smaller the Mw, the greater the DH and the higher the content of polar and non-polar amino acids. This is because, in the process of enzymatic hydrolysis, the peptide bond was decomposed by the action of the protease, and amino acids and small peptide fragments were continuously released into the enzymatic hydrolysis system, resulting in a large increase in the polar and non-polar group content.

### 3.2. Proliferation Inhibition Effect of SPPHs on MGC-803 Cells

**Figure 2:**
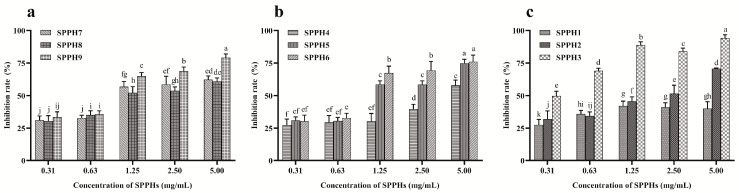
The proliferation inhibition of SPPHs on MGC-803 cells. (**a**) IR of SPPH7, SPPH8 and SPPH9; (**b**) IR of SPPH4, SPPH5 and SPPH6; (**c**) IR of SPPH1, SPPH2 and SPPH3. Each bar represents the mean ± standard of at least three separate experiments. All the data were analyzed by Duncan’s multiple comparisons; bars with no letter in common are significantly different at the level of *p* < 0.05.

The proliferation inhibition effect of SPPHs on MGC-803 cells was analyzed by CCK-8 assay. The IR was examined when the MGC-803 cells were treated with different concentrations (0.31, 0.63, 1.25, 2.50 and 5.00 mg/mL) of SPPHs for 24 h. As shown in Figure 2, SPPHs reduced the number of viable cells in a dose-dependent manner, and produced a 49.76 ± 3.60% reduction (*p* < 0.05) when the cells were treated with SPPH3 (MWCOs < 3k, 0.31 mg/mL). In order to avoid the influence of osmotic pressure or cytotoxicity, just the concentration below IR50 was used to carry out the follow-up experiment. According to the proliferation inhibition results, there was no significant correlation between the DH of the SPPHs and the number of viable cells. As shown in Figure 2a,b, the Mw of the SPPHs had a significant effect on the number of viable MGC-803 cells. The IR was the highest when the cells were treated with a SPPH fraction passed through a 3k Da MWCO membrane.

However, a certain degree of fluctuation was found in the proliferation inhibition effect of SPPHs on MGC-803 cells. Apoptosis plays an important role in cell physiological changes. However, it might lead to a fluctuation of IR if the organelles such as mitochondria still have biological activity. The above results showed that SPPHs might be a novel efficient therapeutic peptide drug to inhibit the proliferation of MGC-803 gastric cancer cells. The compounds of SPPHs were so complex that they may contain different proteins, peptides and amino acids. The amino acid content of hydrolysates with different DH was preliminarily analyzed. In subsequent experiments, we found that the hexapeptide fragment with molecular weight 679.45 Da played a major role (detailed structural analysis data not shown).

### 3.3. Effect of SPPHs on the Morphology of MGC-803 Cells

#### 3.3.1. Inverted Microscope Observation Results

**Figure 3:**
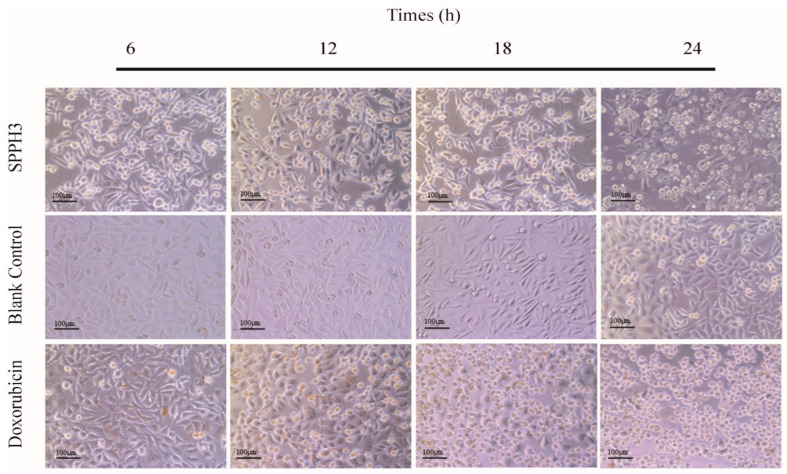
Cell morphology observed by an inverted microscope after treatment with SPPH3 and Doxorubicin at different times. MGC-803 cell proliferation inhibited by SPPH3 (0.31 mg/mL) was visualized; Doxorubicin (0.001 mg/mL) treatment was used as a positive control group. Normal cells were used as a blank control group. All of the cells were observed after 6, 12, 18 and 24 h treatments. The images were captured by a Leica inverted microscope at ×200 magnification. Scale bar = 100 μm.

Phase contrast microscopy was used to visualize the morphological changes produced after treatment with SPPH3 or Doxorubicin or no treatment. As shown in Figure 3 (blank control group), the MGC-803 cells were spindle shaped, with clear cell outlines and smooth surfaces showing a high refractive index. With the extension of the incubation time (treated after 24 h), little protrusions of cells and more intracellular granules could be seen while growing into a dense monolayer with contact inhibition.

When the cells were treated with SPPH3 (0.31 mg/mL), a notable decrease in the cell number accompanied by a change of cell shape and large granules inside the cytoplasm could be clearly observed in the cell morphology (Figure 3, SPPH3). With the extension of the incubation time (treated after 12 h), a notable slenderness of the cells was observed in the cell morphology accompanied by a smaller volume of cells with a low refractive index. Their pseudopodia became shorter and black granules or vacuoles appeared in the cytoplasm. Some circular bodies could be seen around the cells, which were crescent shaped masses or gathered on one side of the cells. After 24 h of incubation, the visual field of the microscope turned cloudy, but adjusting the fine focus of the microscope, just a few normal cells could be seen. Most of the cells were round or oval, large granules were found inside the cytoplasm and cells were cleaved into bright and round corpuscles.

Doxorubicin, an anti-tumor drug, was used as the positive control group (0.001 mg/mL). As shown in Figure 3 (Doxorubicin), after culturing for 12 h, cells began to crack with decreased adhesion, and the floating necrotic cells were increased because the membrane was broken. Cell debris and content were distributed in the culture medium and almost no normal cells were found after 24 h.

#### 3.3.2. Fluorescence Microscope Observation Results

In the early stage of apoptosis, phosphatidylserine (PS), which is distributed in the inner side of the cell membrane, can be turned to the outside of the cell membrane to promote coagulation and inflammation [20]. Annexin V, which is labeled with a fluorescent probe, FITC, can be selectively combined with PS [21]. Annexin V-FITC can be used to detect the important feature of apoptosis directly with a fluorescence microscope. Propidium iodide (PI) can stain necrotic cells, late apoptotic cells which lose the integrity of the cell membrane [21]. An Annexin V kit used with flow cytometry can be used to confirm the proportion of normal cells, early apoptotic cells, apoptotic cells and necrotic cells, and to determine whether the cells are undergoing apoptosis.

**Figure 4:**
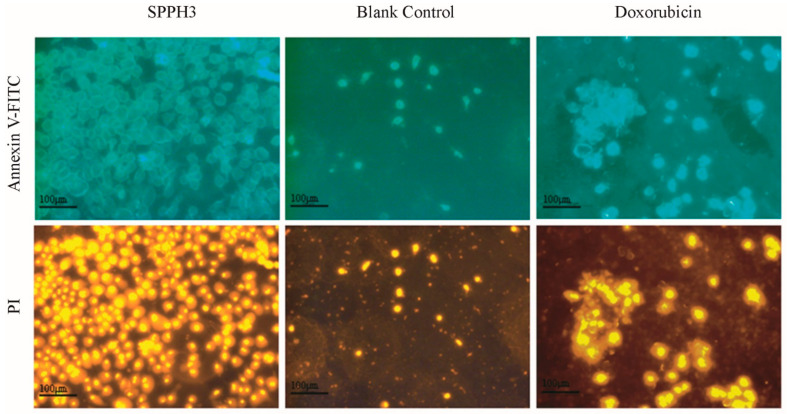
Cell morphology observed by a fluorescence microscope after treatment with SPPH3 and Doxorubicin. Fluorescence microscope images of MGC-803 cells incubated without (Blank Control) or with SPPH3 (0.31 mg/mL) or Doxorubicin (0.001 mg/mL) for 24 h, respectively. Cells were stained with Annexin V-FITC and PI. Images were captured by a fluorescence microscope at ×200 magnification. Scale bar = 100 μm.

The treatment with SPPH3 or Doxorubicin resulted in an increase in floating cells, and cell debris was visibly apparent. Both Annexin V-FITC and PI staining and fluorescence microscopy were used to observe the cell membrane changes of the MGC-803 cells (Figure 4). Blank-control-treated cells remained little-stained because of intact cell membranes. The integrity of the cell membrane was destroyed while those cells were treated by SPPH3 and Doxorubicin. More cells were homogeneously stained and the shape of the cells was irregular. Moreover, Doxorubicin was superior to SPPH3. However, severe damage (such as cell death or cell apoptosis) led by Doxorubicin could be observed in the normal cells (data no shown). It was hypothesized that SPPH3 promoted the apoptosis of MGC-803 cells.

#### 3.3.3. LSCM Observation Results

The cytoskeleton is the network of protein fibers in eukaryotic cells, which not only plays an important role in external forces, maintaining cell morphology and internal structure, but is also involved in other important life activities, such as cell attachment, migration, proliferation and apoptosis [22]. The apoptotic changes of cells labeled with immunofluorescence antibodies can be analyzed conveniently by LSCM.

**Figure 5:**
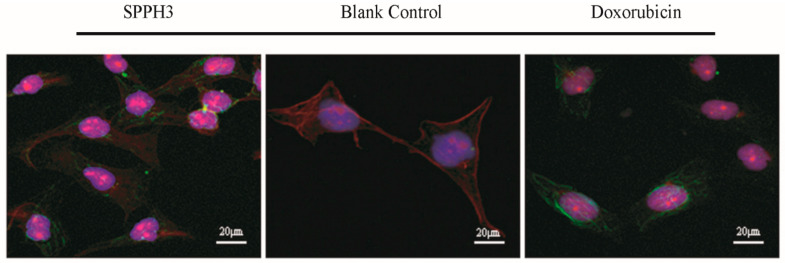
Cell morphology observed by LSCM after treatment with SPPH3 and Doxorubicin. LSCM images of MGC-803 cells incubated without (Blank Control) or with SPPH3 (0.31 mg/mL) or Doxorubicin (0.001 mg/mL) for 24 h, respectively. Cells were processed for indirect immunofluorescence assay to visualize the cytoskeleton which was stained for actin filaments (red), microtubules (green) and nuclei (blue). Images were captured by an LSCM at ×630 magnification. All three stains are superimposed. Scale bar = 20 μm.

DAPI staining and immunofluorescence were used to observe the nuclear and cytoskeleton morphological changes of MGC-803 cells. As shown in Figure 5, blank control cells remained intact and homogeneously stained while those treated with SPPH3 or Doxorubicin showed nuclear fragmentation. Compared with the blank control group, the microtubules of cells treated with SPPH3 and Doxorubicin were stained and diffused inside and outside the cells. The disorganization of microtubules and obvious modification of bundle structures of actin could be observed. Moreover, nuclear fragmentation was found with a change of nuclear morphology (Figure 5). Consequently, the alteration of the actin filaments and microtubule was regarded as loss of the cell membrane integrity. Either SPPH3 or Doxorubicin play a role in the morphology changes of cytoskeleton.

### 3.4. Apoptosis and Cycle of MGC-803 Cells

The fluorescence intensity of PI is in direct proportion to the content of DNA. If the fluorescence intensity of G_0_/G_1_-phase cells is assumed to be 1, the theoretical value of fluorescence intensity of G_2_/M-phase cells is 2. Then, cell cycle and apoptosis analysis can be carried out based on the distribution of DNA content.

With the objective to provide evidence that alteration of the cell cycle progression was responsible for the inhibition of cell proliferation, the DNA content of cells treated with SPPH3 or Doxorubicin by flow cytometry. As shown in Table 1, after treating MGC-803 cells with SPPH3 or Doxorubicin, a significant alteration of the cell cycle progression could be observed. An apparent accumulation in cells in the G_0_/G_1_-phase to 45.17 ± 2.20% (*p* < 0.05), and a decrease in cells in the S-phase to 21.25 ± 4.24% (*p* < 0.05) were observed when MGC-803 cells were treated by SPPH3 (0.31 mg/mL) for 24 h. There was no significant correlation between the DH change of the SPPHs and the ratio of apoptotic cells. However, a significant accumulation in cells in the G_0_/G_1_-phase to 38.82 ± 2.05% (*p* < 0.05) and in the S-phase to 36.79 ± 1.73% (*p* < 0.05), and a decrease in cells in the G_2_/M-phase to 24.76 ± 3.71% (*p* < 0.05) were observed when MGC-803 cells were treated by Doxorubicin (0.001 mg/mL) for 24 h. The apoptotic effect of SPPH3 (0.31 mg/mL) could reach 61.63 ± 0.68%. It indicated that the apoptotic effect of SPPHs was obvious, and that the blocking effect of the SPPHs on the MGC-803 cells was mainly in the G_0_/G_1_-phase.

## 4. Discussion

All the α-amino acids have different side-chains or R groups attached to the α-carbon atom, and so they can be classified as polar amino acids and non-polar amino acids on the basis of their side-chains [23]. Polar amino acids include Arg, His, Asp, etc. Non-polar amino acids include Leu, Try, etc. Peptides or hydrolysates containing different polar and nonpolar groups often show different biological activities such antioxidant and anti-tumor activities. In Zou, T.-B’s point of view [24], the important factor for peptides with antioxidant activity was that their molecules contain a high proportion of hydrophobic amino acids (non-polar amino acids). The common amino acids in antioxidant peptides were hydrophobic amino acids (His, Trp, Phe, Pro, Gly, Lys, Ile and Val) and aromatic amino acids (Trp, Tyr and Phe), which is consistent with our observations. Hydrophobic amino acids at the C-terminal and N-terminal also seemed to contribute to the antioxidant properties of active peptides. The SPPHs prepared by Khammuang, S et al. [25] were proved to protect DNA from hydroxyl radical damage and the active polypeptides purified from their hydrolysate, AKPGVY and AAEYPA, had a good antioxidant effect. Xia, L et al. [26,27] described a silkworm active peptide (amino acid sequence: MNFAKILSFVFALVALSMTSAAPEPRWKIFKKIKMGRNIRDGIVKAGPAIEVLGSAKAIGK), whose polar amino acid was positively charged and could form specific amphiphilic α-helices that interacted with the nonpolar lipid cell membrane and induced cytoskeleton destruction of esophageal cancer cells, and had no toxicity to some normal cells. This evidence demonstrates that amino acid polarity is an important factor to evaluate the activities of hydrolysates and peptides.

At present, there are many studies on the stimulation mechanisms of protein hydrolysates or polypeptides on cells or animals [2,28,29,30]. Generally speaking, the relative molecular weight of these hydrolysates was large, they were difficult to absorb directly and they could only enter the cell by endocytosis [31]. Another way may be to stimulate specific receptors on the surface of target cells to achieve the corresponding chemical signal transmission [32,33]. Li, X.T et al. [9] found that SPPHs could specifically inhibit the proliferation of human gastric cancer SGC-7901 cells and cause abnormal morphological characteristics in a dose-dependent and time-dependent manner through the internal apoptotic pathway. Such results were similar to our results in this study. On the other hand, some researchers have also tried to explore the mechanism of silkworm pupa protein on oxidative damage and the apoptosis of colon cancer DLD-1 cells from the perspective of the mitochondrial metabolism, the glycolysis rate and the energy metabolism and made some progress [2]. In recent years, some studies [34] have gradually developed from proliferation inhibition in vitro to the establishment of tumor-bearing mice or tumor model mice in an attempt to further improve the theory of proliferation inhibition of SPPHs and peptides in vivo. Inhibiting the proliferation and inducing apoptosis of tumor cells in vitro and in vivo could provide experimental evidence for the potential application of SPPHs as a new anti-tumor candidate drug for the treatment of gastric diseases.

For the morphological observation of MGC-803 cells, we used an inverted microscope, a fluorescence microscope, LSCM, a transmission electron microscope (TEM; the corresponding results were published in a previous paper [1]). In fact, it was a gradual research process from physical morphology to cell substructure. Morphological changes of apoptosis were generally multi-stage, natural apoptosis often involved only a single cell, and the apoptosis of a few cells might occur asynchronously [35]. Unless it was due to the intervention of active substances, there would not be concentrated apoptosis or necrosis.

After the treatments with SPPHs, MGC-803 cells appeared to firstly shrink in size, shorten or even lose pseudopodia, and gradually separate from the surrounding cells. Then, for the cell substructure, the increase in cytoplasmic density, the disappearance of mitochondrial membrane potential, the change in permeability, the release of cytochrome c to the cytoplasm, the concentration of nucleoplasm, the fragmentation of nucleoli in the nuclear membrane, and the degradation of DNA were the main characteristics [34]. The cell membrane had vesicular formation, the PS inside the membrane everted to the surface of the membrane, and the cell membrane structure was still intact [20], which were also similar to the results observed by a fluorescence microscope in our study. Finally, the remains of apoptotic cells are divided and wrapped into several apoptotic bodies until the apoptotic bodies are quickly swallowed by the surrounding phagocytes [21]. For the study of the cytoskeleton, we mainly analyzed the root cause of cytoskeleton collapse in morphology. Because actin is a structural protein of microfilaments and a major cytoskeletal protein, it plays an important role in many subcellular processes, such as cytoplasmic flow, organelle and nuclear localization, cell morphogenesis and cell division [36]. Similarly, microtubules, as a polar cytoskeleton, mainly play a role in maintaining cell morphology and assisting intracellular transport, and can be assembled with other proteins into spindles, grana, centrioles, flagella, ciliated neural tubes and other structures [37,38]. The observation of microfilaments and microtubules by LSCM can determine whether the structure of cellular protein fiber network is still intact, and whether the maintenance of morphology, cell movement, intracellular material transport and cell division are normal. Of course, now, there are more advanced technologies such as cryo-electron microscopy, which can conduct in-depth research on biological macromolecules and explain the mechanism in another dimension.

Apoptosis or programmed cell death is a physiological process of cell suicide, which is crucial to the healthy growth of the body or the maintenance of homeostasis [39,40]. The cell cycle consists of four different phases, including two gap phases, G_1_ (the early stage of G_1_-phase is specially called G_0_-phase) and G_2_, as well as the S-phase and M-phase. The processes at different stages of the cell cycle are controlled by several classes of cyclin-dependent kinases (CDKs) [41]. The effects of SPPHs on cell growth found in this study were mainly manifested in the blocking effect of the G_0_/G_1_-phase and the promotion of cell apoptosis. Bioactive peptides (ACBP) extracted from goat spleens showed inhibition activities on the proliferation of human gastric cancer line BGC-823 in vitro; the results of the apoptosis and cell cycle were similar to our current research. Then, at the molecular level, ACBP induced the expression of tumor suppressor and apoptosis genes and inhibited the expression of tumor-promoting genes. In vivo, ACBP significantly inhibited the growth of human gastric tumors in xenotransplantation models [42]. Likewise, chickpea peptides could induce the arrest of the S and G_2_ phases of the Ishikawa cell cycle, DNA fragmentation, down-regulation of Bcl-2 expression, up-regulation of Bax expression and promotion of caspase-3 activation, leading to cell apoptosis [42,43]. Other antimicrobial peptides found in the hemolymph of the Cecropia moth (Hyalophora cecropia) in North America could kill HL-60 leukemia cells through a pro-apoptotic effect, with minimal impact on non-tumor 3T3 fibroblasts [44]. Currently, the anti-tumor properties of natural peptides might not be effective and selective enough to make these compounds usable in human body. However, they provided a basis for further development and modification to improve their efficacy as anti-tumor compounds.

## 5. Conclusions

In summary, our research showed that SPPHs had good proliferation inhibition activity against MGC-803 cells in vitro. The inhibitory effect increased in a dose-dependent manner. The changing of microstructures (cell membrane, nucleus and cytoskeleton) of MGC-803 cells under the treatment with SPPHs also led to the emergence of apoptosis. It also caused the cell cycles to be blocked in G_0_/G_1_-phase. Therefore, SPPHs could be used as a potential anti-tumor substance, which is worth exploring for its mechanism of action and structure–activity relationship in the future. However, the current research results only provide a possibility for in vivo research. There were still some limitations in our research, which need to be further discussed: the digestion and absorption rate of SPPHs in human body, the evaluation of the therapeutic effect of the tumor-bearing model, the structure–activity relationship of SPPHs, the measurement of the dose–effect relationship and so on. Some ethical issues might be involved in this process, which required more institutions with the qualification to participate in the research so that the problem could be solved. Going forward, these also offer important and exciting areas of investigation that may provide new therapeutic perspectives in tumor and disease progression and restoration of homeostasis.

## Figures and Tables

**Table 1:** Effects of different concentrations of SPPHs on cycle distribution and apoptosis rate on MGC-803 cells.

Group	Concentration (mg/mL)	Cells were Treated with SPPHs for 24 h			
		G_0_/G_1_	S	G_2_/M	Apoptosis Rate
Blank	-	34.41 ± 0.74 ^d^	31.51 ± 1.46 ^b^	33.52 ± 1.16 ^a^	2.95 ± 0.65 ^d^
Doxorubicin	0.001	38.82 ± 2.05 ^c^	36.79 ± 1.73 ^a^	24.76 ± 3.71 ^b^	ND
SPPH1	0.31	41.26 ± 2.24 ^bc^	23.83 ± 1.54 ^c^	34.74 ± 3.28 ^a^	42.23 ± 2.61 ^b^
SPPH2	0.31	40.24 ± 1.36 ^c^	24.41 ± 9.42 ^c^	33.51 ± 0.11 ^a^	30.07 ± 4.74 ^c^
SPPH3	0.02	38.73 ± 0.72 ^c^	24.35 ± 1.49 ^c^	36.97 ± 1.92 ^a^	32.80 ± 2.21 ^c^
SPPH3	0.04	42.25 ± 1.73 ^ab^	21.46 ± 4.09 ^c^	36.77 ± 3.10 ^a^	35.13 ± 5.50 ^c^
SPPH3	0.08	42.54 ± 0.88 ^ab^	25.80 ± 3.38 ^c^	32.23 ± 3.36 ^a^	36.70 ± 7.54 ^bc^
SPPH3	0.16	43.06 ± 0.80 ^ab^	25.55 ± 1.26 ^c^	32.28 ± 1.15 ^a^	59.20 ± 0.53 ^a^
SPPH3	0.31	45.17 ± 2.20 ^a^	21.25 ± 4.24 ^c^	33.77 ± 2.44 ^a^	61.63 ± 0.68 ^a^

“-” means zero dose. “ND” means none detected. The data are expressed as the mean ± standard deviation from triplicate experiments (unit = %). Values with no letters in common in each column are significantly different (*p* < 0.05).

## Data Availability

Data is contained within the article.
